# LIG4 Syndrome Presenting with Extensive Cutaneous Viral Warts Caused by Multi-Type HPV Infection

**DOI:** 10.3390/genes17080917

**Published:** 2026-08-03

**Authors:** Kaichen Tang, Shiqi Fan, Rui Zhang, Miao Sun, Dong-Lai Ma, Xue Zhang

**Affiliations:** 1 McKusick-Zhang Center for Genetic Medicine, State Key Laboratory for Complex Severe and Rare Diseases, Institute of Basic Medical Sciences & School of Basic Medicine, Chinese Academy of Medical Sciences & Peking Union Medical College, Beijing 100005, China; tkc19@student.pumc.edu.cn (K.T.); fanshiqi@pumch.cn (S.F.); b2025005002@student.pumc.edu.cn (R.Z.); xuezhang@pumc.edu.cn (X.Z.); 2 Department of Dermatology, Peking Union Medical College Hospital, Chinese Academy of Medical Sciences and Peking Union Medical College, National Clinical Research Center for Skin and Immune Diseases, Beijing 100730, China

**Keywords:** severe combined immunodeficiency, human papillomavirus infection, warts, DNA ligases

## Abstract

**Background**: DNA Ligase 4 (LIG4) catalyzes the final ligation step during V(D)J recombination. Biallelic pathogenic variants in LIG4 cause severe combined immunodeficiency (SCID), a life-threatening immunodeficiency characterized by the lack of mature T and B cells. **Case Presentation**: An 18-year-old male exhibited widespread verrucous and keratotic cutaneous lesions across the face, neck, and extremities. The proband manifested early-onset short stature, chronic malnutrition, recurrent pulmonary infections with bronchiectasis, chronic diarrhea, and bilateral tenosynovitis. Immunophenotyping revealed persistent panhypogammaglobulinemia, absent B cells, reduced T and natural killer (NK) cells, and nearly undetectable naïve T cells. Both α-HPV and β-HPV were detected in the cutaneous lesions. Compound heterozygous LIG4 variants (hg19, NM206937.2, c.833G > T; p.Arg278Leu inherited from the mother; c.833G > A; p.Arg278 His inherited from the father) were identified. **Conclusions**: This study reports a proband with LIG4 syndrome presenting with rare phenotypes of multiple viral warts and combined lymphopenia of T, B, and NK cells. This proband harbors compound heterozygous variants at the same codon (c.833G), which not only confirm the clinical diagnosis but also enrich the known phenotypic spectrum of LIG4 syndrome.

## 1. Introduction

Inborn errors of immunity (IEIs) are a group of complex diseases characterized by compromised immunity and increased risks of infection, autoinflammation, autoimmunity, and/or malignancy [1]. V(D)J recombination is the process by which T and B lymphocytes generate a diverse repertoire of antigen receptors. Defects in this process give rise to severe combined immunodeficiency (SCID), a critical subtype of IEI, usually characterized by the absence of mature T and B cells [2]. Pathogenic variants in genes vital for the key stages of V(D)J recombination, including the cleavage phase (*RAG1*, *RAG2*, *NUDCD3*), end processing (*DCLRE1C*), ligation and repair (*LIG4*, *NHEJ1*, *XRCC4*, *PRKDC*), and genome surveillance (*ATM*, *MRE11A*, *RAD50*, *NBN*, *RNF168*), all led to SCID [2].

Non-homologous end joining (NHEJ) mediates primary DNA double-strand break (DSBs) repair [3]. The NHEJ components LIG4 and XRCC4 interact to complete the final DNA ligation [4]. Physiological DSBs usually arise during V(D)J recombination, where LIG4 governs the essential ligation step for functional rearrangement [5]. LIG4 deficiency is an extremely rare autosomal recessive IEI caused by pathogenic variants in *LIG4*. Affected patients present with a broad clinical spectrum dominated by developmental retardation and SCID [6], usually accompanied by recurrent severe infections and shortened lifespan. LIG4 deficiency exhibits prominent phenotypic heterogeneity, with lymphopenia being the most striking clinical feature.

In this case report, we reported a proband with SCID carrying compound heterozygous variants at the same codon in *LIG4*. The proband displayed disseminated viral warts, short stature, chronic malnutrition, recurrent pulmonary infections with bronchiectasis, chronic diarrhea, and bilateral tenosynovitis. To the best of our knowledge, this is the fourth reported case of widespread viral warts complicating LIG4 syndrome. Our findings further support the possibility that *LIG4* variants contribute to the development of widespread viral warts and broaden the phenotype spectrum of SCID caused by variants in *LIG4*. Our case offers valuable insights into the risk factors underlying cutaneous HPV infection in LIG4 syndrome.

## 2. Materials and Methods

### 2.1. Study Oversight

Clinical information and samples were collected from the family trio, and informed consent was obtained. This study was approved by the Peking Union Medical College Institutional Review Board in accordance with the Declaration of Helsinki. (Approval No: zs2024016).

### 2.2. Genetic Testing

Genomic DNA was extracted with the QIAamp DNA Blood Mini Kit (QIAGEN, 51106, Hilden, Germany). Libraries were sequenced on Illumina NovaSeq X Plus (Illumina, San Diego, CA, USA) at 100× depth, and reads were aligned to GRCh37/hg19 using BWA (https://bio-bwa.sourceforge.net/). Variants were filtered under an autosomal recessive inheritance model. We retained variants with sequencing depth ≥ 20 bp, and excluded common variants (allele frequency ≥ 1%) from gnomAD v4.1.1, ExAC, 1000 Genomes, and NHLBI-ESP. Only exonic nonsynonymous variants and splice-altering variants predicted by SpliceAI (https://spliceailookup.broadinstitute.org/#) were kept. Variants classified as pathogenic by at least one tool (SIFT, Polyphen, MutationTaster, CADD v1.7) were selected, and their frequencies were rechecked in gnomAD v4.1.0. All candidate variants were validated by PCR and Sanger sequencing. The primers for PCR and Sanger sequencing were *LIG4*-front primer: 5′-TGGAAAAAGTCTGTAGGCAACTG-3′; and *LIG4*-reverse primer: 5′-GCCATCATCTCACCATCAAG-3′. For phenotype-based analysis, we further analyzed variants in genes responsible for T-B-SCID and viral warts. Pathogenic genes associated with T-B-SCID, EV, and viral warts were listed in Supplementary Table S1.

### 2.3. Genotyping of HPV

Sterile swabs were taken from skin lesions, and genomic DNA was extracted using the TIANGEN DP322-02 kit (Tiangen Biotech, Beijing, China). A total of 1 μg of DNA was sheared into ~350 bp fragments with a Covaris sonicator (LE2202-plus, Woburn, MA, USA), and library quality was assessed by AATI (5400). High-quality reads were aligned to the host genome via Bowtie2 (http://bowtie-bio.sourceforge.net/bowtie2/index.shtml) to filter out host sequences. The remaining reads were de novo assembled using MEGAHIT (https://github.com/voutcn/megahit, accessed on 29 June 2026), and scaffolds were split at ambiguous N bases to generate N-free scaftigs. Open reading frames were predicted from scaftigs longer than 500 bp using MetaGeneMark (http://topaz.gatech.edu/GeneMark/); sequences below 100 nt were removed. Redundant sequences were clustered with CD-HIT (http://www.bioinformatics.org/cd-hit/) to build an initial non-redundant gene catalog. Clean reads were mapped to this catalog using Bowtie2, and genes covered by ≤2 reads per sample were discarded to obtain the final Unigene set. Gene abundance was calculated based on mapped read counts and gene length, followed by basic statistics, core/pan genes, and sample correlation analyses. All Unigenes were finally annotated against the PaVE database for HPV genotyping.

## 3. Results

### Clinical Presentation

**Figure 1:**
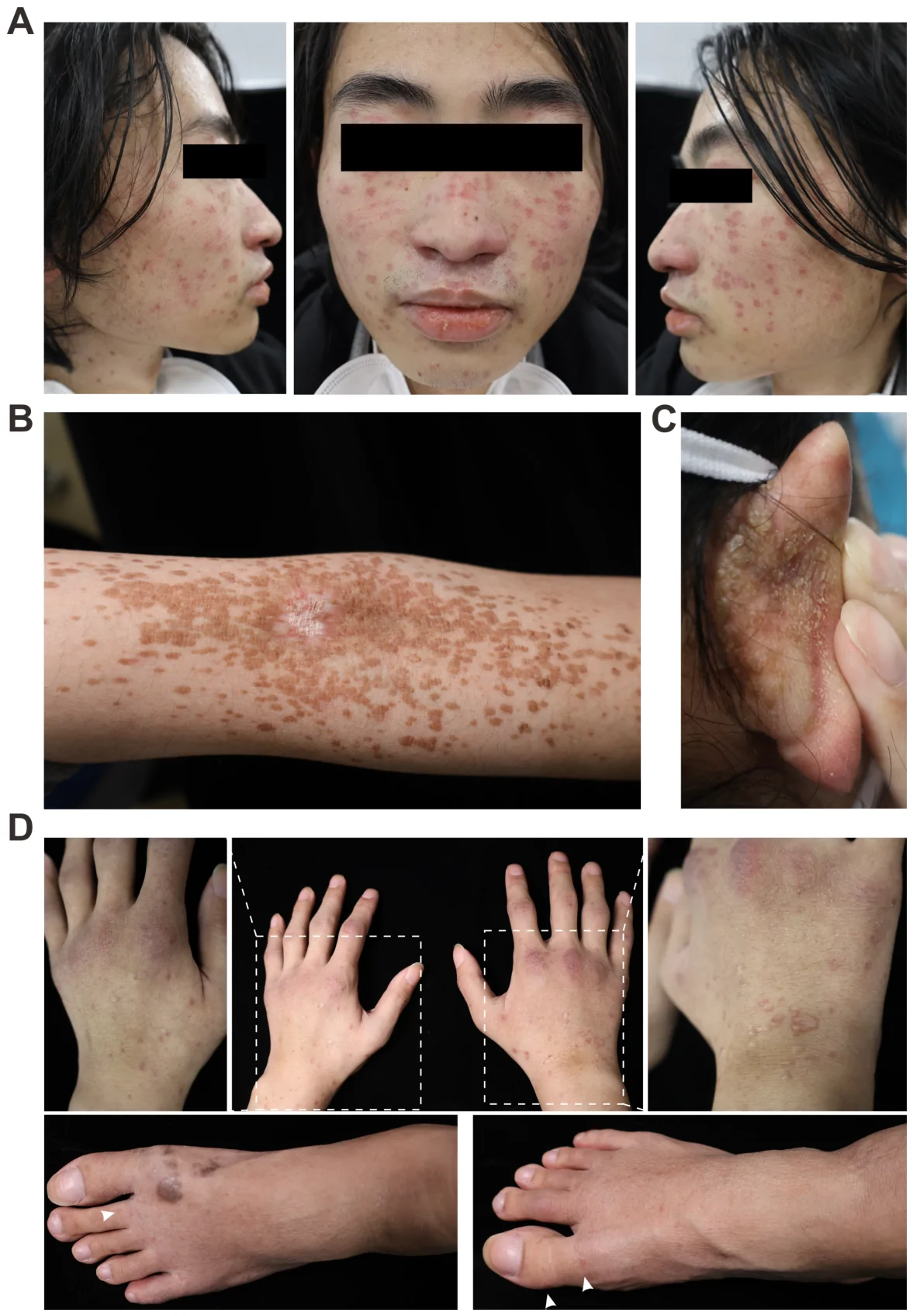
Clinical imaging of the proband. Panel (**A**) shows the skin lesions on the proband’s face. Panel (**B**) demonstrates numerous warts on the proband’s antecubital fossa. The lesions were similar in both antecubital fossae and popliteal fossae. Panel (**C**) shows keratotic lesions behind the proband’s ear. The lesions were neither painful nor pruritic. Similar skin lesions were observed on the contralateral ear. Panel (**D**) shows the proband’s extremities. The white arrows indicate planar warts on the proband’s feet.

An 18-year-old male presented to the dermatology clinic with a 1-month history of disseminated skin lesions. Initially, scattered erythema appeared on the proband’s nose and both cheeks (Figure 1A). The proband was initially diagnosed with systemic lupus erythematosus (SLE), as the skin lesions resembled a butterfly rash. However, the skin lesions rapidly spread to the entire face, neck, and extremities, accompanied by elevated planar wart-like lesions. Numerous scattered verrucous lesions were noted on both the cubital fossae and the popliteal fossae. Additionally, cutaneous keratosis and desquamation were observed behind both ears. (Figure 1B–D). Metagenomic sequencing of skin swabs from the lesions revealed a large number of low-risk HPV sequences (Supplementary Table S1). Among these genotypes, HPV78 is the only α-HPV and is capable of inducing flat warts. Based on the proband’s cutaneous manifestations, a diagnosis of immunodeficiency and generalized verrucosis was made. The majority of the identified HPVs were β-HPVs, among which HPV49 represents an epidermodysplasia verruciformis (EV)-associated genotype, suggesting a possible EV diagnosis.

The proband was short for his age and had experienced long-standing malnutrition since early childhood. Apart from this, he had been healthy with no notable medical problems prior to 10 years of age. At the age of 10 years, he began to experience recurrent severe pulmonary Pseudomonas aeruginosa infections. Chest CT revealed bronchiectasis. The proband was hospitalized for severe pulmonary infections about every six months. Additionally, he developed chronic diarrhea. Two years ago, the proband presented with swelling, pain, and limited motion in the bilateral metacarpophalangeal joints and first interphalangeal joints, accompanied by morning stiffness lasting approximately half an hour. Ultrasound revealed tenosynovitis of the extensor tendons in the dorsal aspects of both wrists. Autoantibodies, including ANA, anti-dsDNA, anti-RNP, anti-Sm1, anti-SSA, anti-SSB, anti-Scl-70, anti-Jo-1, anti-MPO, and anti-PR3, were negative.

The proband exhibited persistent panhypogammaglobulinemia in repeated testing at ages 9, 17, and 18. The complete blood count (CBC) results indicate severe lymphopenia, accompanied by thrombocytopenia. Immunophenotyping of peripheral blood showed absent B cells, with decreased T and NK cell counts. Naïve CD4 and CD8 T cells were almost absent (Supplementary Table S2). Bone marrow aspiration revealed hypocellular marrow with decreased megakaryocytes. EBV and CMV DNA in peripheral blood were undetectable. Stool culture was positive for *Salmonella typhimurium* (*S. typhimurium*). Tests for HIV, syphilis, HBV, and *Mycobacterium tuberculosis* were all negative. No factors suggestive of secondary immunodeficiency were identified.

**Figure 2:**
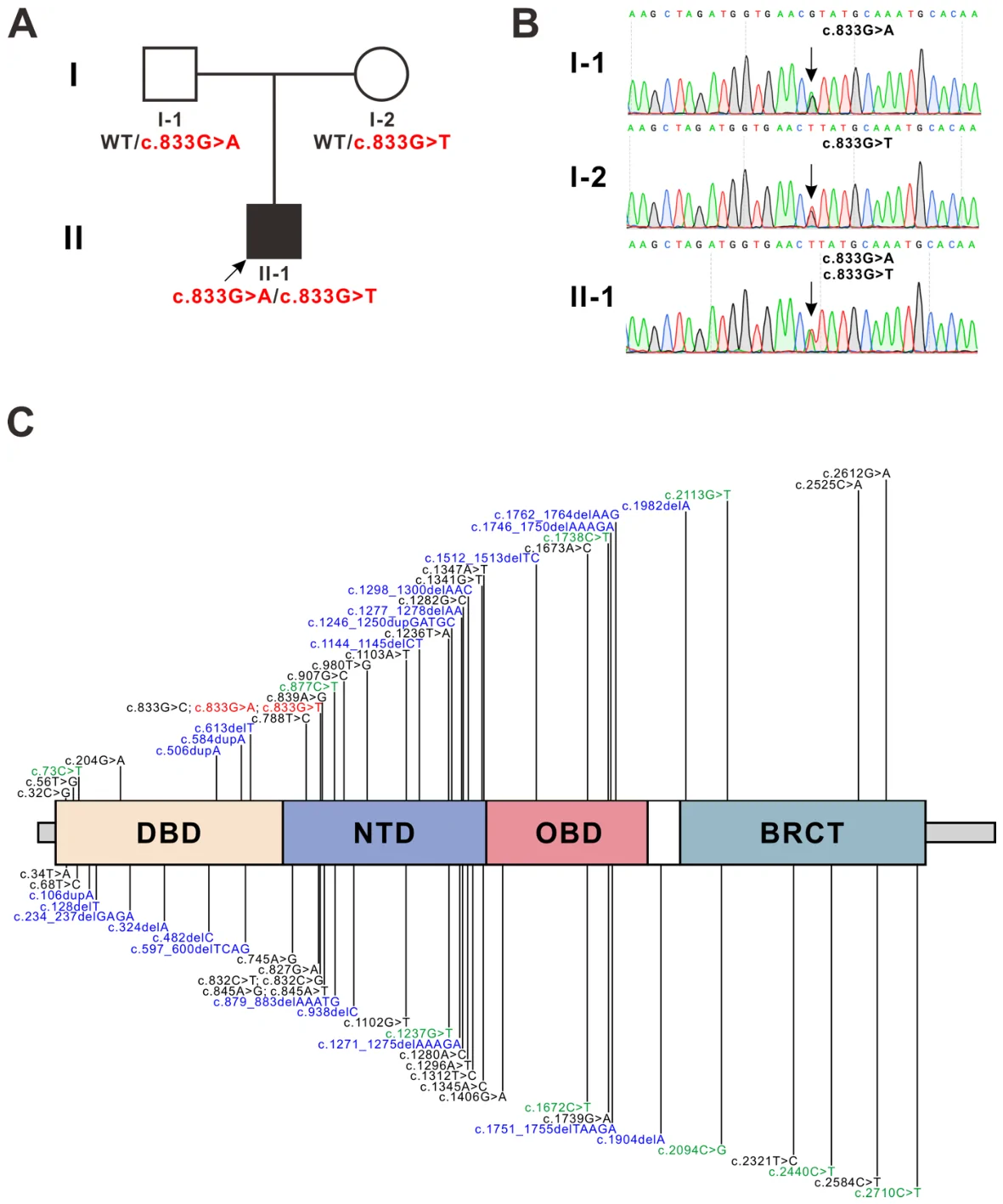
Genetic analysis of the pedigree. Panel (**A**) shows the pedigree. The proband is indicated by an arrow. Solid black denotes affected individuals. Panel (**B**) presents the Sanger sequencing results. The pathogenic variant site is indicated by the black arrow. Panel (**C**) illustrates the mutational spectrum of LIG4 in the Human Gene Mutation Database (HGMD). Black indicates missense variants. Green represents nonsense variants. Blue represents indel variants. Red highlights the proband’s variant. DBD, DNA-binding domain; NTD, nucleotidyltransferase domain; OBD, oligo-binding domain; BRCT, BRCA1 C-terminal domains.

Given the severe reduction in both T and B cells, the proband was suspected of having a T-B- severe combined immunodeficiency (SCID). Therefore, Trio-WES was performed on the proband and his parents to establish a definitive diagnosis. Compound heterozygous variants in *LIG4* (hg19, NM_206937.2, c.833G > T; p.Arg278Leu inherited from the mother; c.833G > A; p.Arg278 His inherited from the father) were identified via Trio-WES (Figure 2A,B). The proband harbors biallelic compound heterozygous variants in *LIG4* at the same codon, located in the critical nucleotidyltransferase domain (NTD) of *LIG4* (Figure 2C). These variants were previously reported and proved to be pathogenic [7,8]. No other candidate pathogenic variants were detected in known SCID-related genes. To further explore whether viral warts arose from additional genetic variants, we screened for pathogenic variants underlying EV. Variants within all previously reported EV-related genes were further analyzed, yet no suspicious pathogenic alterations were identified beyond the *LIG4* variants. On the basis of clinical manifestations and genetic findings, a final diagnosis of LIG4 syndrome was established for the proband.

## 4. Discussion

In this study, we reported a case of LIG4 syndrome with the rare phenotypes of multiple viral warts and combined lymphopenia of T, B, and NK cells. This proband harbors compound heterozygous variants at the same codon in *LIG4* (hg19, NM_206937.2, c.833G > T; p.Arg278Leu inherited from the mother; c.833G > A; p.Arg278 His inherited from the father). These variants are located in the NTD of LIG4 and are highly evolutionarily conserved and vital for LIG4 catalytic ability. The p.Arg278Leu variant is highly recurrent in the Chinese LIG4-deficient population and exhibits a distinct founder effect [7]. To date, nearly 90 causative variants in *LIG4* have been reported, most of which are indels and nonsense variants that lead to defective protein expression (Figure 2C).

LIG4 syndrome represents a clinically heterogeneous spectrum ranging from classical SCID to milder immunodeficiency with prominent radiosensitivity and cancer predisposition [5]. Immunological disturbances of LIG4 deficiency typically result in profound T- and B-cell lymphopenia, hypogammaglobulinemia, and extreme susceptibility to severe, recurrent infections. Despite the severe immunological profile, the proband survived into adolescence without hematopoietic stem cell transplantation.

Epidermodysplasia verruciformis (EV) (OMIM #226400) is a genetic disorder characterized by impaired immunity against cutaneous low-risk HPV, predominantly β-HPV, which results in uncontrolled cutaneous HPV infection and extensive viral warts. EV is regarded as a subset of generalized verrucosis. The diagnosis of EV should be established upon detection of EV-specific HPV types [9]. In contrast, generalized flat warts are mostly acquired, caused by low-risk α-HPV, self-limited, and without malignant potential. EV is classified into typical and atypical subtypes based on the presence or absence of systemic immunodeficiency phenotypes. Although the molecular mechanism of EV remains to be fully elucidated, studies have indicated that impaired T-cell function is a key mechanism underlying the development of atypical EV [10]. Prior to this study, three patients with LIG4 syndrome have been reported to present with disseminated viral warts, suggesting that *LIG4* is a disease-causing gene associated with atypical EV [6,7,11].

Viral warts confined to the popliteal and antecubital fossae rather than to sun-exposed regions are extremely rare. Risk factors for cutaneous HPV infection complicating LIG4 syndrome may include genetic background, sex, lifestyle, nutritional status, and sun exposure. Among these, specific *LIG4* variant sites and variants in EV-associated genes are likely to play important roles in the genetic background. Among the three previously reported patients with LIG4 syndrome and viral warts, only one underwent NK cell evaluation and showed reduced NK cell counts. The patient developed viral warts on both hands and feet at the age of eight, and was found to carry c.833G > T, p.R278L, and c.1271_1275delAAAGA, p.K424RfsX20 variants [7]. Previous studies have highlighted that NK cell function is critical for immunity against high-risk HPV. Furthermore, restoration of NK cell function via hematopoietic stem cell transplantation can effectively alleviate cutaneous lesions [12]. The other patient carried three homozygous *LIG4* variants (8C > T, A3V; 26C > T, T9I; 833G > A, R278H) [6]. The third patient carried compound heterozygous c.1237G > T, E413X, and c.1341G > T, W443C. Although three of the four patients carried the R278 variant, not all patients with this variant developed warts. Male sex may also be a potential risk factor. Of the four patients, three were male, and the sex of the fourth was not specified.

## 5. Conclusions

In summary, this case strengthens the evidence that *LIG4* variants can underlie cases manifesting a viral wart phenotype. To the best of our knowledge, this is the first study to determine the genotype of HPV in viral warts in patients with LIG4 syndrome based on metagenomic sequencing analysis. Defective T cells and NK cells may coordinately contribute to severe viral infection.

## Data Availability

All datasets are included in the manuscript or in the Supplementary Material.

## Supplementary Materials

The following supporting information can be downloaded at https://www.mdpi.com/article/10.3390/genes17080917/s1, Table S1: HPV detected in skin lesions. Table S2: Laboratory Examinations.

## Abbreviations

The following abbreviations are used in this manuscript:

IEI	Inborn errors of immunity
V(D)J	Variable-(diversity)-joining
SCID	Severe combined immunodeficiency
DSB	DNA double-strand break
NHEJ	Non-homologous end joining
EV	Epidermodysplasia verruciformis
HPV	Human papillomavirus
α-HPV	Alphapapillomavirus
β-HPV	Betapapillomavirus
γ-HPV	Gammapapillomavirus
SLE	Systemic lupus erythematosus
CT	Computed tomography
CBC	Complete blood count
NK	Natural killer
EBV	Epstein–Barr virus
HIV	Human immunodeficiency virus
HBV	Hepatitis B virus
Trio-WES	Trio whole-exome sequencing
NTD	Nucleotidyltransferase domain
HGMD	Human Gene Mutation Database
DBD	DNA-binding domain
OBD	Oligo-binding domain
BRCT	BRCA1 C-terminal domains
PaVE	Papillomavirus Episteme
HSCT	Hematopoietic stem cell transplantation
WT	Wild type
IUIS	International Union of Immunological Societies
